# The Immediate and Long-Term Impact of Military Aircraft Noise on Hearing: A Cross-Sectional Comparison of Fighter Pilots and Ground Staff

**DOI:** 10.3390/ijerph18062982

**Published:** 2021-03-14

**Authors:** Chao-Yin Kuo, Chia-Lien Hung, Hsin-Chien Chen, Cheng-Ping Shih, Rou-Huei Lu, Chen-Wai Chen, Li-Wen Hung, Yi-Chun Lin, Hang-Kang Chen, Da-Ming Chu, Yuan-Yung Lin, Yueh-Chun Chen, Chih-Hung Wang

**Affiliations:** 1 Department of Otolaryngology-Head and Neck Surgery, Tri-Service General Hospital, National Defense Medical Center, Taipei 11490, Taiwan; chefsketchup@hotmail.com (C.-Y.K.); acolufreia@yahoo.com.tw (H.-C.C.); eric660802@gmail.com (C.-P.S.); mushroom263527@gmail.com (R.-H.L.); liwen1976@gmail.com (L.-W.H.); hwalongchen@yahoo.com.tw (H.-K.C.); yking1109@gmail.com (Y.-Y.L.); 2 Institute of Cognitive Neuroscience, National Central University, Taoyuan 32001, Taiwan; 3 Taichung Armed Forces General Hospital, Taichung 41168, Taiwan; chia-lien@803.org.tw; 4 School of Public Health, National Defense Medical Center, Taipei 11490, Taiwan; ifroo29@gmail.com (C.-W.C.); chudaming@yahoo.com.tw (D.-M.C.); 5 Graduate Institute of Medical Sciences, National Defense Medical Center, Taipei 11490, Taiwan; lyc_1023@yahoo.com.tw

**Keywords:** military noise, noise-induced hearing loss (NIHL), fighter aircraft, pilot, ground staff, pure-tone audiometry (PTA), extended high-frequency (EHF) audiometry, distortion-product otoacoustic emissions (DPOAEs), signal-to-noise ratio (SNR)

## Abstract

We examined the immediate and long-term impacts of military aircraft noise exposure on noise-induced hearing loss (NIHL) in fighter pilots and ground staff. We recruited 40 pilots, 40 ground staff, and 136 age-matched controls; all participants underwent hearing tests, including conventional pure-tone audiometry (PTA) (0.25–8.0 kHz), extended high-frequency (EHF) audiometry (9.0–18.0 kHz), and distortion-product otoacoustic emission (DPOAE) as a recent reference. A subsequent hearing test immediately after flight-mission noise exposure was requested. The results revealed higher recent hearing thresholds in pilots and ground staff than in controls. Threshold shifts at many octave band frequencies were also significantly elevated in ground staff. The grouped frequency threshold was significantly elevated in the 4–8 kHz high-frequency range. After a single flight-mission noise exposure, both ground staff and pilots showed decreased signal-to-noise ratios for DPOAE (1–8 kHz), whereas only ground staff showed significantly elevated left-ear hearing thresholds at 3, 11.2, and 12.5 kHz by conventional and EHF PTA. Fighter pilots and ground staff serve in hazardous noise-exposed environments that cause hearing damage and subsequent NIHL, but ground staff may be more vulnerable. A comprehensive hearing conservation program should be implemented to protect high-risk service members, and especially ground staff, from high-intensity noise exposure.

## 1. Introduction

The adverse impact of noise from military operations on hearing among military service members has long been considered an important global health issue. The resulting hearing impairment can also further interfere with the effective execution of military operations. Hearing loss is one of the most common occupational health hazards, and tinnitus and hearing impairment are the most common disabilities among veterans of the United States military forces [1,2]. 

Noise can be classified into different types, including continuous, variable, intermittent, or impulsive, depending on how the noise changes over time. Continuous noise may cause greater cochlear damage and a permanent hearing threshold shift compared to intermittent noise [3]. Impulse noise, which is characterized by less than 1 s of high-intensity sound with sharp rise and decay, is even more pernicious in its damage compared with continuous sound with the same acoustic power spectrum [4]. With compulsory military service gradually shifting to a volunteer force, the risk of occupational noise-induced hearing loss (NIHL) in people serving in the military workplaces should be recognized and prevented. 

Several clinical auditory functional tests are used currently, but pure-tone audiometry (PTA) has long been a useful and widely used tool for assessing auditory function for hearing screening in both the clinical and on-site workplace settings. However, otoacoustic emissions (OAEs) have subsequently been introduced for research and clinical applications [5,6]. The OAEs represent the amplifications of sounds originating from the outer hair cells (OHC) in the cochlea and can be measured in the external auditory canal [6,7]. Clinical applications of OAEs therefore mainly focus on the identification of sensory hearing loss due to outer hair cell damage. The OHCs are the first and the most affected cells in NIHL [8], so an OAE examination provides a quick and easy way to monitoring hearing status in settings like the military [9] or industry [10,11]. Furthermore, the decrease in the amplitude of the distortion product otoacoustic emissions (DPOAEs) is also well correlated with OHC loss [12]. Compared to other types of OAEs, the DPOAEs seem to be superior for predicting the development of NIHL [13,14]. 

Another hearing test, extended high-frequency (EHF) audiometry, is now drawing much interest for early detection of sensorineural hearing loss. EHF audiometry is defined as a threshold measurement above the frequency of 8 kHz and is especially useful for detecting damage at the basal turn of the hair cells. Several studies have suggested that EHF audiometry is particular useful for assessing ototoxic drug-induced and noise-induced hearing loss, as well as age-related hearing loss (presbycusis) associated with sub-clinical hearing difficulties [15,16,17,18]. 

The aim of the present study was to use these audiometric examination tools to survey the hearing status of young military personnel serving at an air force base as a way to establish the best diagnostic tool for NIHL detection. A secondary aim was to identify the immediate noise impact of a single flight mission on the hearing of fighter pilots and air force ground staff. 

## 2. Materials and Methods

### 2.1. Study Design and Recruitment of Participants

This cross-sectional study evaluated the hearing condition of military personnel before and after a one-time noise exposure during flight missions. We recruited military members who served at a military air base, enrolling ground staff, fighter aircraft pilots, and age-matched control subjects whose duties did not involve fighter aircraft operation. Participants with a history of ear trauma, any previous ear surgery, or otitis media were excluded. All participants completed a questionnaire regarding their daily work, hearing condition, and whether they routinely wore a hearing protection device (HPD) in or over the ears while exposed to hazardous noise levels exceeding 85 dB within the workplace. Before performing the hearing tests, all participants underwent otoscopic examinations by an otologist to ensure the patency of the external auditory canal and to exclude any otological diseases. The recent hearing conditions of personnel in each group, reflecting the long term impact of military noise on hearing, were determined by conventional PTA, EHF audiometry, and DPOAE tests. Pilots and ground staff who were exposed to a single flight task-related noise were requested to undergo another hearing test battery as above within 2–4 h after the exposure. 

### 2.2. Ethical Considerations

The Tri-Service General Hospital Institutional Review Board approved the research project with ethical approval code: TSGHIRB 2-105-05-107.

### 2.3. Conventional Pure-Tone and Extended High-Frequency Audiometry

Participants were tested in a sound-attenuating test booth established in a van equipped with a mobile audiologic unit that conformed to ANSI/ASA S3.1-1999 (R2018) specifications for audiometric test rooms. We obtained conventional PTA of all subjects at octave frequencies from 0.25 to 8 kHz for each ear using a Grason-Stadler GSI 61 clinical audiometer (Nicolet Biomedical, Madison, WI, USA) with TDH-50P headphones (Telephonics Corp., Farmingdale, NY, USA). Frequencies over 8 kHz are termed extended high-frequencies (EHFs). Pure-tone hearing thresholds in the EHF range (9, 10, 11.2, 12.5, 14, 16, and 18 kHz) were determined using a GSI 61 clinical audiometer with Sennheiser HDA 200 circumaural earphones (Sennheiser Co, Germany). A descending⁄ascending method in 5 dB steps revealed hearing threshold levels. Responses were considered reliable if the difference between the test and retest thresholds was no more than 5 dB. If thresholds exceeded the equipment’s maximum output level, a “Χ” symbol was indicated. 

We calculated the arithmetic mean of the grouped-frequencies threshold as pure-tone averages (PTAs) of three-frequency (0.5, 1, and 2 kHz; 3FPTA), four-frequency (0.5, 1, 2, and 4 kHz; 4FPTA), high-frequency (4, 6, and 8 kHz; HFPTA), overall-frequency (0.25, 0.5, 1, 2, 3, 4, and 8 KHz; OFPTA), and extended high-frequency (9, 10, 11.2, 12.5, 14, 16, and 18 kHz; EHFPTA) PTAs. 

### 2.4. Otoacoustic Emissions

Participants were tested in a sound-attenuating test booth, as described previously for obtaining the PTA. The distortion product otoacoustic emissions (DPOAEs) were recorded for both ears of all participants using the Bio-Logic Scout Sport System (v. 3.02, model 3.33.00, Natus Medical Inc., Schaumburg, IL, USA). The DPOAE test consisted of presenting two primary tones at frequencies f1 and f2 and levels L1 and L2. The frequency ratio f2/f1 was fixed at 1.22. The stimulus sound pressure levels were set at L1 = 65 dB and L2 = 55 dB. These parameters are expected to elicit optimal DPOAE magnitudes [19,20]. Insert transducers were placed in each participant’s ear canal, where the paired primary tones were delivered. Distortion product OAEs corresponding to the frequency 2f1-f2 were recorded and plotted as a function of f2 frequency (DP-grams). The DPOAE level was compared against the noise floor estimate for the corresponding center frequency to determine the signal-to-noise ratio (SNR). For all f2 frequencies, only SNR > 6 dB was regarded as a presented DP that could be included for comparing the change between tests.

### 2.5. Noise Measurement and Analysis

The noise level was measured and recorded from within the hardened aircraft shelter, where the fighter aircraft is protected and the ground staff can perform necessary maintenance and prepare the aircraft for flight. For acoustic measurements, the equipment included an acoustic fixture (GRAS 45CB; Sound & Vibration AS, Denmark) and a blast probe microphone (GRAS 67SB) operated with power support modules (GRAS 12AA 2-Channel Power Module, SysCheck generator and GRAS 12AQ 2-Channel Universal Power Module). This equipment was placed 7 m from the aircraft, as this setting simulated the real working distance between the ground staff and aircraft and met ANSI S12.42 standards. Computers (Lenovo Ideapad 700 15ISK) equipped with SpectraPlus-DT and SpectraPlus-DT9837A software (Pioneer Hill Software LLC. Poulsbo, WA, USA) were used for data collection and analysis. All equipment was calibrated using the GRAS Type 42AP Intelligent Pistonphone. Application of fast Fourier transform gave a noise spectrum and spectrogram that showed the distribution of noise intensities as a function of frequency over time.

### 2.6. Data Analysis

The statistical differences among the groups were analyzed using the Kruskal-Wallis nonparametric ANOVA. Results for the rate of HPD wearing were compared with the chi-squared test or Fisher’s exact test. Results of audiograms before and after flight mission noise were compared with a paired t-test. An independent t-test compared the audiograms between the pilots and ground staff. All statistical analyses were conducted using SPSS software, Version 22.0 (IBM, Armonk, NY, USA), and a *p*-value less than 0.05 was considered statistically significant.

## 3. Results

### 3.1. Characteristics of Participants

A total 216 participants were recruited for this study: 40 ground staff, 40 fighter pilots, and 136 age-matched control subjects. Male participants accounted for 91% (*n* = 196) of the participants, reflecting the significant gender difference not only in this study (*p* < 0.002) but also in military air bases in general. Although control subjects were recruited with the criteria of no history of working in a high A-weighted equivalent SPL places (> 85 dB), the habit of HPD wearing on the air base was also investigated. No significant statistical difference was detected among the three groups in terms of HPD wear or between the ground staff and fighter pilot groups (Table 1). 

### 3.2. The Recent Hearing Thresholds Were Significantly Higher for Ground Staff Than for Fighter Pilots

**Figure 1:**
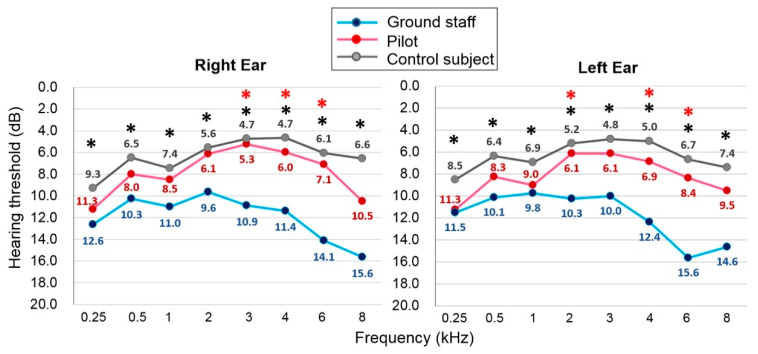
Recent audiometric outcomes assessed with conventional pure-tone audiometry. The value of each data point indicates the average threshold across subjects at the corresponding frequency. Comparisons between groups were performed using the Kruskal—Wallis test, followed by Scheffe’s post-test. * black asterisk indicates *p* < 0.05 when comparing the ground staff group with the control subjects. * red asterisk indicates *p* < 0.05 when comparing the ground staff group with the pilot group.

The conventional PTA test showed that the recent hearing thresholds were highest in the ground staff, followed by the fighter pilots, when compared to the control group. Although both the pilot and ground staff groups had higher hearing thresholds than the control group, only the ground staff showed significantly elevated hearing thresholds compared to the control, and this was evident at all frequency bands (*p* < 0.05; Figure 1). In addition, the hearing thresholds at 3, 4, and 6 kHz for the right ear, and at 2, 4, and 6 kHz for the left ear were significantly higher in the ground staff group than in the pilot group (*p* < 0.05; Figure 1). No characteristic 4 kHz notch was evident in the audiometric profiles of the ground staff and pilot groups; however, their elevated hearing threshold showed deterioration starting at a frequency of 4 kHz. Use of the grouped frequencies PTA for comparison revealed that both the 4FPTA (*p* = 0.038 for right ear and *p* = 0.034 for left ear) and HFPTA (*p* = 0.025 for right ear and *p* = 0.029 for left ear) that involved a high frequency greater than 4 kHz showed significantly higher threshold averages in the ground staff than in the fighter pilots (Table 2). These data suggest that aircraft noise may have greater auditory insults at a high frequency range of 4–8 kHz. 

**Figure 2:**
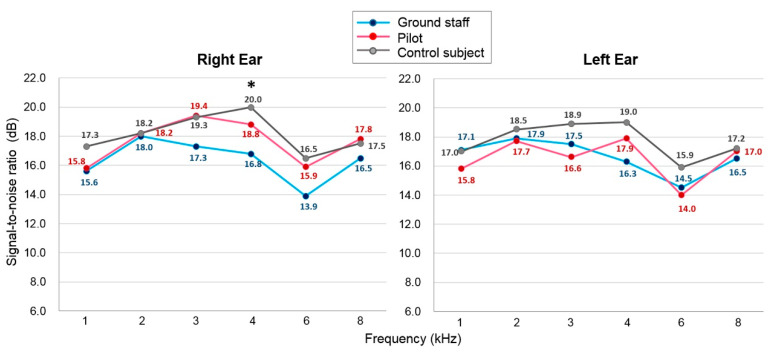
Recent signal-to-noise ratios (SNR) of the distortion-product optoacoustic emissions in the three groups. The value of each data point indicates the average SNR across subjects at the corresponding frequency. * indicates *p* < 0.05, compared between the ground staff and control subjects.

The SNR values of the DPOAEs were greater than 6 for all frequencies in the right and left ears of all three groups; therefore, all the SNR values were considered acceptable responses. Only the 4 kHz frequency in the right ear of the ground staff group showed a significantly decreased SNR value compared to the value for the pilots and the control subjects (Figure 2). A trend showing a greater decrease in decreased SNR values was apparent in the ground staff group compared to the other two groups.

### 3.3. The EHF Audiometry Metric Showed a Statistically Significant Group Difference

**Figure 3:**
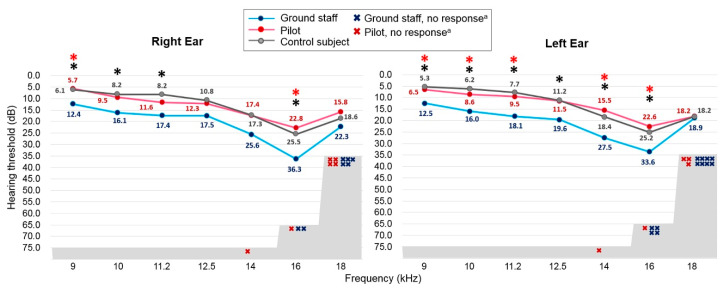
Average extended high-frequency thresholds measured from frequencies of 9–18 kHz in the ground staff and pilot groups. The value of each data point indicates the average thresholds across subjects at the corresponding frequency. Gray areas indicate the thresholds detected beyond the maximum output of the audiometer. Hearing thresholds that fell into a gray area were drawn with red “X” symbols for pilots and blue “X” symbols for ground staff. Comparisons between the groups were performed using the Kruskal—Wallis test, followed by Scheffe’s post-test. * black asterisk indicates *p* < 0.05 when comparing the ground staff with the control subjects. * red asterisk indicates *p* < 0.05 when comparing the ground staff group with the pilot group.

The average hearing thresholds at frequencies from 9 to 18 kHz measured by EHF audiometry demonstrated a similar trend as that observed with the conventional PTA, as the threshold values were more elevated in the ground staff group than in the pilot group. The pilot group did not show any significantly higher threshold than the control group, whereas the ground staff had significantly higher hearing thresholds than the control subjects in the following frequency bands: 9, 10, 11.2, and 16 kHz for the right ear (*p* < 0.05), and 9, 10, 11.2, 12.5, 14, and 16 kHz for the left ear (*p* < 0.05, Figure 3). Statistically significant elevations of hearing thresholds were also evident at 9 and 16 kHz in the right ear (*p* < 0.05), and at 9, 10, 11.2, 14 and 16kHz in the left ear of the ground staff group compared to the pilot group (*p* < 0.05, Figure 3). 

Comparison of the total number of “X” symbols in both groups revealed that the number was larger for the ground staff than for the pilot groups. However, the number of “X” symbols at 14, 16, and 18 kHz showed no significant difference between the groups using Fisher’s exact test for comparison (Table 3). The overall comparison of elevated hearing thresholds using the EHF PTA at 9–18 kHz revealed a trend toward differentiation between the ground staff and pilot groups (*p* = 0.060). These data also suggest a greater vulnerability to noise injury among the ground staff at the air base than among the pilots.

### 3.4. Immediate Noise Impact on Hearing after Accomplishing One Flight Mission

**Figure 4:**
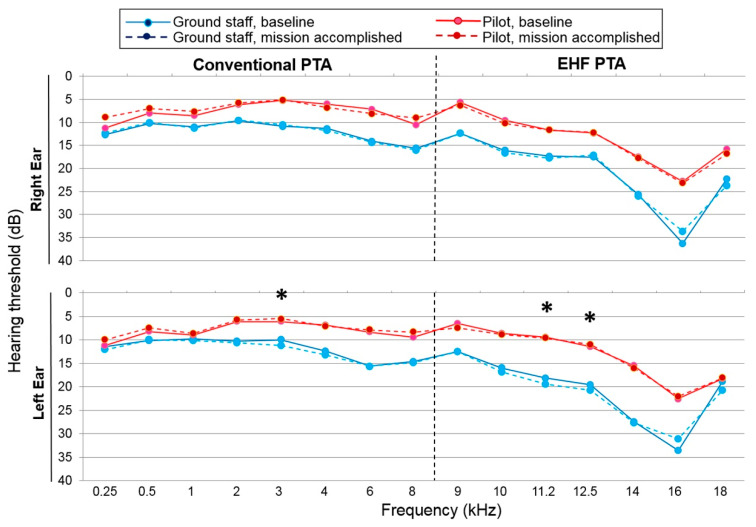
Mean of the conventional and EHF pure-tone audiometric hearing thresholds for both ears for the ground staff and pilot groups before and after flight mission execution. * *p* < 0.05 when comparing the mean hearing thresholds before and immediately after the flight mission in the ground staff using an independent t-test. PTA = pure-tone audiometry; EHF = extended high-frequency.

The use of conventional PTA and EHFPTA revealed that almost all the octave frequencies from 0.25 to 18 kHz in both groups showed few changes after the ground staff and pilot groups completed a single flight mission. Only three significantly elevated threshold shifts were observed at the frequencies of 3 (1.3 ± 0.5 dB; *p* = 0.016), 11.2 (1.4 ± 0.7 dB; *p* = 0.047), and 12.5 kHz in the left ears (1.1 ± 0.5 dB; *p* = 0.018) of the ground staff group (Figure 4). No significantly elevated threshold changes were observed in the pilot group after noise exposure from the flight mission. 

**Figure 5:**
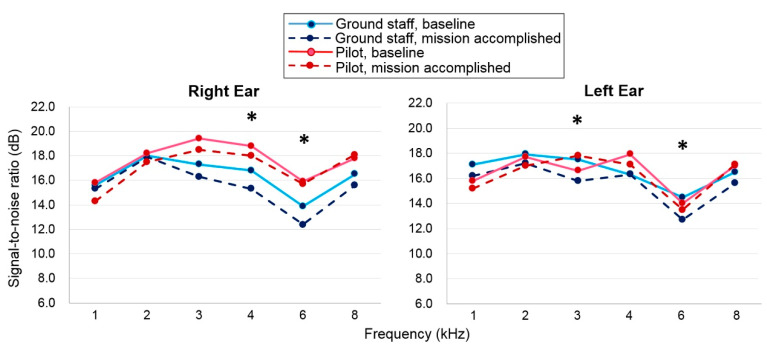
Mean of the signal-to-noise ratio of distortion-product otoacoustic emissions (DPOAEs) for both ears between the ground staff and pilot groups before and after a flight mission execution. * indicates *p* < 0.05, comparing the ground staff group before and after the flight mission noise exposure.

The use of DPOAEs revealed that the average SNR values of the DPOAEs in both groups decreased at nearly all frequencies from 1–8 kHz after noise exposure. The SNR after flight mission was significantly lower in ground staff group at frequencies of 4 kHz (−1.5 ± 0.7 dB; *p* = 0.03) and 6 kHz (−1.5 ± 0.6 dB; *p* = 0.01) in the right ear and at 3 kHz (−1.7 ± 0.6 dB; *p* = 0.01) and 6 kHz (−1.8 ± 0.6 dB; *p* = 0.009) in the left ear (Figure 5). On the contrary, the pilot group showed no significant changes in the SNR level after the flight mission. These data indicated that DPOAEs are more sensitive to subtle cochlear insults than are the conventional PTA readings.

### 3.5. Analysis of the Noise Level in in the Hardened Aircraft Shelter

The hardened aircraft shelter is one of the noisiest work sites on the military air base, and is where the ground staff spent most of their working hours per day. We were therefore interested in using the blast probe microphone and the acoustic test fixture to investigate the physical characteristics of the noise. The blast probe microphone collected the original signals in the sound field, which represents the original physical characteristics of the noise. By contrast, the acoustic test fixture simulates the presence of a person with the physical characteristics of the human auditory system, head, torso, and pinna to provide the head-related transfer function (HRTF); therefore, it could contribute a further acoustic gain and present a relatively realistic recapitulation of the noise impact on an individual’s hearing system. 

During a one-hour recording and measurement by the blast probe microphone, the measured equivalent SPL was 85.3 dB. The sound levels that exceeded 90% (LA90), 50% (LA50), and 10% (LA10) of the measurement period were 93.2, 123.4, and 124.1 dB, respectively. Conversely, the use of the acoustic test fixture for recording gave a measured equivalent SPL of 115.3 dB, and the LA90, LA50, and LA10 of the measurement period were 123.0, 156.6, and 157.4 dB, respectively. These data indicated that the hardened aircraft shelter is a hazardous and noisy work environment and that the ground staff require HPD appropriate to their practical needs.

**Figure 6:**
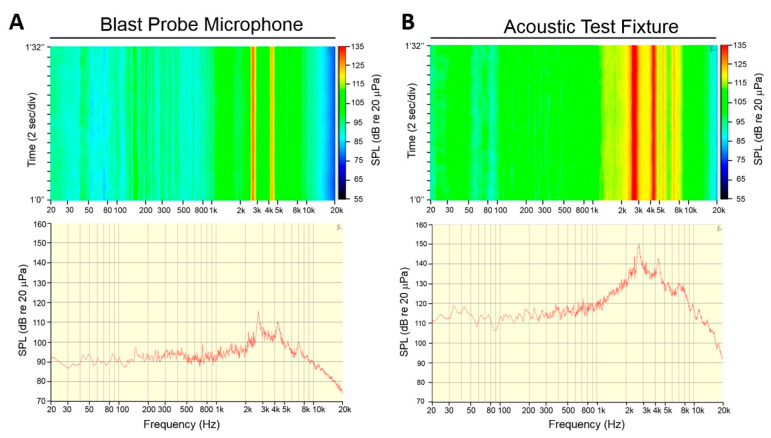
Spectrogram and power spectrum of the noise inside the hardened aircraft shelter for 32 s, collected by (**A**) the blast probe microphone (GRAS 67SB) and (**B**) the acoustic test fixture (GRAS 45CB). SPL = sound pressure level.

**Figure 7:**
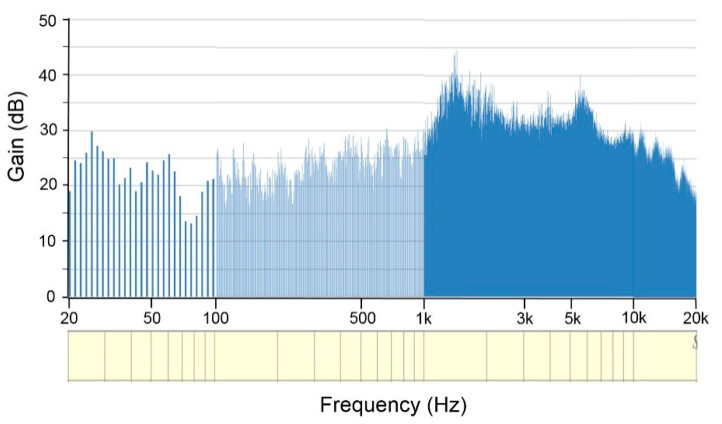
Transfer function of the acoustic test fixture shows the gain estimated from the data collected by the blast probe microphone.

Figure 6A shows the spectrogram and power spectrum of the noise measured within a duration of 32 s by the blast probe microphone; the continuous and steady characteristics of this noise is indicated by the perfectly straight lines of noise energy distributed in the frequency domain. Loud noises over 100 dB were largely located across the range of frequencies from 2–5 kHz. Figure 6B shows the spectrogram and power spectrum of the noise measured by the acoustic test fixture within the same time frame. The measured sound pressure level across 2–5 kHz can exceed 130 dB. The power spectrum revealed two further peaks: the first one (150.3 dB) appeared at 2716 Hz and the second one (142.5 dB) at 4275 Hz (Figure 6B). These data indicated that the frequency involved in this high-intensity noise within the hardened aircraft shelter tended to cross over the range of 2.5 to 4.5 kHz. The transfer function of the acoustic test fixture that mimics the human pinna and the external auditory canal could provide a further mean acoustic gain of 26.8 dB (13.0–44.4 dB) (Figure 7).

## 4. Discussion

One of the main goals of this research was to examine the prevalence and severity of hearing loss in military personnel serving on an air force base. Surprisingly, the mean pure-tone averages from 0.25–8 kHz in each group were within normal limits (<20 dB), not to mention the characteristic noise notch at 4 kHz demonstrated in the audiogram. A focus on the overall trend of the age distribution of recruited participants revealed that 77.5% of the ground staff and 90% of the pilots were approximately 35 years old, whereas 92.5% of the ground staff and 97.5% of the pilots were under 40 years old. In other words, the vast majority of the participants in this study might have had a relatively shorter duration of noise exposure than had occurred in the other studies that recruited members who had over 20 years of military service [21,22]. The recent PTA results in our study were compatible with those from a previous large scale investigation in U.S. Air Force aviation-related personnel by demonstrating that changes in the hearing threshold was small during the first 20 years of an individual’s career and before age 50 [22]. The proportion of permanent threshold shift would increase with increasing age [23]. 

However, comparison of the pure-tone audiograms from the two aircraft groups and the control group still revealed a clear threshold elevation in the pilots and a statistically significant threshold shift (STS) in the ground staff group. Interestingly, both the ground staff and pilot groups showed increases in their hearing thresholds starting at 3 kHz, suggesting that aircraft noise exposure had affected the personnel’s hearing even though the exposure duration was shorter than 20 years. The SNR of DPOAEs of the three groups also revealed a similar trend by showing relatively low SNR values for the ground staff, followed by the pilot values. The EHF audiometry metric may provide more robust evidence to complement the observations obtained with conventional PTA. The higher rate of measurements that were out of the maximum output level of the equipment shown in the recent EHF audiometry of the ground staff, and particularly for the left ear, was consistent with the results obtained using conventional PTA and DPOAEs. 

The conventional PTA and DPOAEs are the most common audiometry methods used for screening and monitoring personnel under the regulations of occupational hearing conservation programs. Both hearing tests are also applied to investigate hearing in military service members who participate in gunfire practice or artillery exercises involving high-intensity impulse noise. Recently, EHF audiometry was reported to provide more information regarding the hearing status covering the high-frequency range from 9 kHz to 20 kHz; therefore, EHF may be able to detect deficits that might be overlooked in noise-exposed populations assessed by conventional PTA [24,25]. The elevated threshold in EHF audiometry and low-level OAEs can both appear before behavioral audiograms present obvious changes; therefore, they may predict susceptibility and sensitive in early diagnosis of NIHL [14,26,27,28], as shown in our study. 

Only limited reports have described the different impacts of aircraft noise on pilots and ground staff. The previous literature mentions a higher-than-limit noise exposure and the vulnerability of ground staff working in civilian airports [29,30], but the hazards to ground staff at military air force bases have not yet been evaluated. One retrospective study that recruited 76 participants (34 helicopter pilots and 42 aircraft mechanics) from the Thai Army also reported that aircraft mechanics suffered from damage to the hearing frequencies involved in speech and high frequencies and a greater decibel loss than the aviators [31]. The difference of noise impact on hearing between ground staff and pilots may reflect several factors, such as the use of hearing protection [31,32], the participant’s smoking status [31,33], the duration of occupational noise exposure, and co-exposures to non-occupational noise or specific chemicals such as organic solvents, welding fumes, carbon monoxide, and hydrogen sulfide [34,35,36]. We also speculate that sonic booms (the sound associated with the shock waves) usually lag behind the aircraft; therefore, the most unbearably loud noise would be encountered by the ground staff, who may spend a further consecutive hour working in the hardened aircraft shelter, either close to or 7 m away from the aircraft, as they conduct the outside inspection and checks before the fighter aircraft leaves the shelter for the runway. The engine is kept running to the whole time and produces significant loud jet noise. By contrast, the pilots are relatively well protected from this noise by the windshield and their helmets and earplugs. A cohort analysis of audiogram records from Air Force aviation-related personnel conducted by Greenwell et al. reported that the age and elapsed time since the baseline audiogram were associated with decreased hearing sensitivity [22]. However, we adequately controlled both age and service duration in our study; therefore, further investigation is needed to identify the cause of this discrepancy and the risk factors that deteriorate hearing in the ground staff specifically. 

Conventional PTA and EHF audiometry revealed statistically significant differences in STS in the ground staff across a wide range of frequencies. By contrast, the recent DPOAEs records showed only one significant decrease in the SNR level at frequency of 4 kHz in the right ears of the ground staff group. Much of the literature has documented that DPOAE amplitudes are poorly correlated with PTA thresholds [35,37]. Interestingly, the tendency for the lowest SNR level to occur in the ground staff, followed by the pilots, seems to be consistent with the comparison of the recent PTA between the groups. Job et al. conducted a 3-year follow-up study on a population of 521 pilots and found that reduced DPOAE levels constitutes a risk for early hearing loss [13]. This finding is also supported by the results in the present study.

The investigation of the immediate impact of noise on the hearing system after only one military flight task was another goal of this study. In agreement with the early findings in the recent PTA values, only the ground staff showed significant temporary thresholds shifts and only at some band frequencies (3 kHz, 11.2 kHz, and 12.5 kHz) in the left ear after flight noise exposure. The pilots showed negligible changes in the PTA values. By contrast, the DOPAE measurements were more sensitive for detection of subclinical impairments than was behavioral tonal audiometry. Again, the significantly reduced SNR level shown by the ground staff indicated that aviation-related personnel may exhibit different vulnerabilities to aircraft noise. The reduction in the SNR may result from louder noise exposure experienced by the ground staff than by the pilots, or the ground staff may be more vulnerable to noise impacts and show greater changes in the SNR than the pilots do after noise exposure. 

Unlike civilian or enterprise factories that follow noise control regulations and receive annual noise survey/auditing, military camp sites usually restrict noise assessment to specific equipment, weapons, exercises, and training. Calculation of the eight-hour time-weighted average sound level (8-hour TWA), by averaging different exposure levels during an exposure period for each person, would provide more fundamental information that could clarify the difference in hearing status among personnel experiencing different noise impacts. However, the real situation for conducting investigations on the military air base may not allow us to survey every possible environment that has extra high-level noise. That is a limitation of our current study. We chose the hardened aircraft shelter as a realistic example of a high-level noise environment because the ground staff spend most of their working hours per day there and because both the ground staff and the fighter pilot would encounter substantial noise during each single flight mission.

Both the fighter pilot and the ground staff are exposed to different types of noise with different intensities, even when they serve in the same workplace—the air base. The recognition of this difference prompted the writing of this article, as we wanted to point out that personnel situated in different noise-exposed environments should have different regulations regarding hearing protection.

To our knowledge, this is the first prospective study that has proposed the possibility of a hearing insult among the pilots and the ground staff during a single flight mission. The results of immediate monitoring of the changes in hearing acuity after a one-off high-intensity noise exposure and the recent hearing status after a long-term noise exposure point to an urgent crisis in the hearing health of military personnel. However, these results also highlight the need for a concerted effort to improve the auditing practice based on current hearing conservation programs that have varying levels of success. 

## 5. Conclusions

Both EHF audiometry and the DPOAE test provide additional information for predicting military aircraft noise that results in NIHL. EHF is more sensitive in detecting potentially lasting NIHL, whereas DPOAEs are more able to reveal the immediate noise impact on hearing. These findings suggest that EHF audiometry is a suitable adjunct to conventional PTA during regular hearing monitoring, while DPOAE is helpful for instantaneous evaluation of noised-induced cochlear insult. A significant hearing threshold shift clearly occurred in both the pilots and the ground staff; therefore, a more comprehensive hearing conservation regulation should be implemented at military air bases to prevent at-risk service members, and particularly aircraft ground staff, from high-intensity noise exposure.

## Figures and Tables

**Table 1:** The distribution of the subjects by group, sex, age, and habit of wearing a hearing protection device (HPD).

	Control Subject (*n* = 136)	Ground Staff (*n* = 40)	Fighter Pilot (*n* = 40)	*p*-Value
**Sex**				<0.002 **
**Male** (%)	130 (95.6)	31 (77.5)	35 (87.5)	
**Female** (%)	6 (4.4)	9 (22.5)	5 (12.5)	
**Age** (Mean ± SD)	31.6 ± 5.2	31.5 ± 7.5	29.2 ± 4.6	0.051
**Wearing HPD**				0.806 ^a^ 0.675 ^b^
**Yes** (%)	125 (91.9)	38 (95.0)	36 (92.3)	
**No** (%)	11 (8.1)	2 (5.0)	3 (7.7)	

** indicates a statistically significance with *p*-value < 0.01; ^a^ results of the HPD wearing rate between the three groups were assessed by the chi-squared (χ²) test; ^b^ results of the HPD wearing rate in the ground staff and fighter pilot groups were assessed by Fisher’s exact test. HPD = hearing protection device; SD = standard deviation.

**Table 2:** Comparison of the arithmetic mean of the grouped frequency threshold between the groups.

Grouped Frequencies (kHz)	Right Ear			Left Ear		
	Ground Staff	Fighter Pilot	*p*-Value	Ground Staff	Fighter Pilot	*p*-Value
	Mean ± SEM	Mean ± SEM		Mean ± SEM	Mean ± SEM	
3FPTA (0.5, 1, 2)	10.29 ± 1.30	7.54 ± 0.90	0.086	10.04 ± 2.38	7.96 ± 1.42	0.159
4FPTA (0.5, 1, 2, 4)	10.56 ± 1.35	7.16 ± 0.89	0.038 *	11.00 ± 1.47	7.25 ± 0.92	0.034 *
HFPTA (4, 6, 8)	13.71 ± 2.25	7.88 ± 1.18	0.025 *	14.21 ± 2.43	8.25 ± 1.06	0.029 *
OFPTA (0.5, 1, 2, 3, 4, 6, 8)	11.94 ± 1.60	7.85 ± 0.93	0.030 *	11.78 ± 1.64	8.19 ± 0.89	0.055
EHFPTA (9, 10, 11.2, 12.5, 14, 16, 18)	19.36 ± 2.73	12.95 ± 1.83	0.056	17.84 ± 2.61	12.47 ± 1.49	0.079

Values are expressed as mean ± SEM. * *p* < 0.05. 3FPTA = three-frequency pure-tone averages; 4FPTA = four-frequency pure-tone averages; EHFPTA = extended high-frequency pure-tone averages; OFPTA = overall-frequency pure-tone averages; EHFPTA = extend high-frequency pure-tone averages; SEM = standard error of the mean.

**Table 3:** Comparison of the rates of output levels beyond the equipment maximum in measuring hearing thresholds using EHFPTA in the ground staff and fighter pilot groups.

Frequency (kHz)	Right Ear			Left Ear		
	Ground Staff	Fighter Pilot	*p*-Value	Ground Staff	Fighter Pilot	*p*-Value
	*n* (%)	*n* (%)		*n* (%)	*n* (%)	
14	0 (0.0)	1 (2.5)	1.000 ^a^	0 (0.0)	1 (2.5)	1.000 ^a^
16	2 (5.0)	1 (2.5)	1.000 ^a^	4 (10.0)	1 (2.5)	0.359 ^a^
18	5 (12.5)	4 (10.0)	1.000 ^a^	8 (20.0)	3 (7.5)	0.105
9–18	5 (12.5)	4 (10.0)	1.000 ^a^	9 (22.5)	3 (7.5)	0.060

^a^ Fisher’s exact test. EHFPTA = extended high-frequency pure-tone averages.

## Data Availability

The data presented in this study are available on request from the corresponding author.
